# Is gene editing an acceptable alternative to castration in pigs?

**DOI:** 10.1371/journal.pone.0218176

**Published:** 2019-06-24

**Authors:** Maria Cristina Yunes, Dayane L. Teixeira, Marina A. G. von Keyserlingk, Maria J. Hötzel

**Affiliations:** 1 Laboratório de Etologia Aplicada e Bem-Estar Animal, Departamento de Zootecnia e Desenvolvimento Rural, Universidade Federal de Santa Catarina, Florianópolis, Brazil; 2 Departamento de Ciencias Animales, Facultad de Agronomía e Ingeniería Forestal, Pontificia Universidad Católica de Chile, Santiago, Chile; 3 Animal Welfare Program, Faculty of Land and Food Systems, The University of British Columbia, Vancouver, British Columbia, Canada; Universidade do Porto Instituto de Biologia Molecular e Celular, PORTUGAL

## Abstract

Male piglets are commonly castrated to eliminate the risk of boar taint. Surgical castration is the commonly used procedure and is known to induce pain. Gene modification targeted at eliminating boar taint in male pigs has been proposed as a possible alternative to surgical castration. The aims of this study were to explore public acceptability of this biotechnology using a mixed methods approach. Quantitative data to assess acceptability of 570 participants from southern Brazil were analysed with multinomial logistic regression models and Spearman correlations; qualitative responses of the reasons provided in support of their position were coded into themes. Just over half of the participants (56%) considered gene modification of male pigs acceptable. Acceptability was lower among participants who grew up in an agricultural environment (ρ = 0.02), but was not influenced by sex, age, religion, urban or rural living, or level of education. Acceptability of gene modification of male pigs as an alternative to surgical castration was positively related to the perception of benefits (r = -0.56, ρ<0.0001) and negatively related to the participant’s perception of risks (r = -0.35, ρ<0.0001). Acceptability was not related to knowledge of basic concepts of genetic biotechnologies (r = 0.06, ρ<0.14), or to awareness of issues related to pig castration or boar taint (r = 0.03, ρ<0.44), both of which were low among participants. Participants that considered gene modification of pigs acceptable justified their position using arguments that it improved animal welfare. In contrast, those that were not in favour were generally opposed to genetic modification. Unforeseen downstream consequences of using genetic modification in this manner was a major concern raised by over 80% of participants. Our findings suggest that perceived animal welfare may encourage public support of gene editing of food animals. However, potential risks of the technology need to be addressed and conveyed to the public, as many participants requested clarification of such risks as a condition for support.

## Introduction

Meat of male pigs that have not been castrated may present an odour and flavour called boar taint, caused by substances that accumulate in the fat (i.e. androstenone and skatole), that many consumers find repulsive [1–3]. To avoid boar taint, most piglets are surgically castrated soon after birth, a known painful procedure; however, pain control measures during and after the procedure are available [4, 5], but not routinely used [6]. An alternative to surgical castration is immunocastration, which is done through a vaccine that induces the production of antibodies against GnRH that inhibits testicular development and function; thereby, reducing fat androstenone concentrations to levels below the reported threshold for human sensory detection [5, 7]. Another option is the production of entire males with acceptable levels of boar taint, through a combination of early slaughter and specific feeding and environmental measures [8]. This option, however, is restricted to some European countries [6] and, in Brazil, the slaughter of uncastrated male pigs is prohibited [9]. Many producers also consider alternative measures to surgical castration or the use of pain control costly, impractical or ineffective [6, 10, 11].

The use of gene editing technology to produce pigs that lack testicular development is a potential alternative that can prevent boar taint [12]. With a gene edition tool, the gene KISSR (responsible for testicular development in pigs) can be knocked-out [12]. Gene editing is a tool that can be more easily and precisely used to manipulate an animals’ genetics compared to previously available technologies. This type of ‘editing’ differs from transgenic technology, which involves insertion of a foreign gene into the genome of an organism, as it may use the species’ own DNA [13]. However, negative attitudes to the use of genetic manipulation in food production [14, 15], especially the animal-based applications [16–19], suggest that the public may not support gene editing of farm animals. With the exception of the salmon in Canada and U.S. [20], no other forms of gene modification in farm animals have been approved for human consumption [21].

Gene editing of animals (and humans) is advancing rapidly and specific applications are already submitted for approval by regulatory bodies, with very little information of public acceptance or understanding of barriers to acceptance. The importance of informing and dialoguing with the public about the use of new biotechnologies in food production is widely recognised [22, 23]. For example, when transgenic products were introduced to the market, it was done under the assumption that the public understood this technology and therefore would accept it; however, surveys indicated a large majority of the public reject them [24, 25]. The prospect of bringing gene edited animals into animal production systems in the near future has stimulated discussion regarding technical and ethical aspects of the technology (e.g. [26–28]).

This is the first direct exploration of public attitudes towards gene editing of pigs. The aim of this study was to explore Brazilian citizens’ acceptability of gene edition of male pigs for prevention of boar taint, perceptions of risks and benefits, and the underlying reasons. A secondary aim was to identify demographic aspects that may influence acceptability.

## Materials and methods

This study was approved by the Ethics Committee on Experimentation of the Santa Catarina State University (P. 2.280.893).

### Participants’ recruitment

Participant recruitment took place at the Hercílio Luz International Airport located in Florianópolis, Santa Catarina, Brazil, a location chosen because of the intense movement of middle-class people [29] and frequent waiting times. People waiting in the public airport hall located before security were approached and invited to participate in the study. To reduce self-selection bias, participants were asked if they would be willing to take a survey about animal production, with no specification of the nature of the issue. Each participant who volunteered received a consent form and was asked to read and sign before taking the survey. Only participants that were at least 18 years old and who permanently resided in one of the three southern states of Brazil (i.e. Rio Grande do Sul, Santa Catarina and Paraná states) and provided signed consent were included in the study. The identity of the participants was not required.

### Description of the survey

Data collection was conducted during the months of September and October 2017. After the first 30 participants had completed the survey their responses were reviewed and refinements made to the questionnaire. The final questionnaire was 5 pages long on single-sided A4 paper and included a total of 24 open and closed questions. Participants took between 10 to 20 minutes to complete the questionnaire. Responses were transferred to the platform QuestionPro (https://www.questionpro.com) and checked for typing mistakes by the first and last author.

The first questions addressed participants’ socio-demographic information relating to whether they lived in one of the three southern states in Brazil, sex (male, female), age (18–25, 26–35, 36–45, 46–55, 56–65, over 66 years old), education (elementary school, high school, or higher education), whether they were religious or not, whether they lived in a rural or urban area, their level of association with livestock production ("not involved"–no involvement in livestock production, "involved"–professional involvement with livestock production (rural producer, student, faculty or profession, etc.), or “grew up in a farming environment”–(family owned a farm or participated in some form of agricultural activity), whether they have visited a pig production farm (yes, no), whether they had ever watched any documentary or video on the Internet, television, etc., showing how a pig farm works (yes, no), their sources of information on animal production (TV—rural programs, Internet, university or school, friends, animal product advertising, non-governmental organizations (NGO) campaigns), and how many times a week they ate meat (none, rarely, 1 to 2 times, 3 to 4 times, 5 to 7 times). Participants that ate meat (even when only rarely) were also asked how important eating meat was for them as an individual (not important, of little importance, intermediate, important, very important), and if they considered the welfare of animals when they ate meat (never, almost never, sometimes, often, always).

Participants were also asked two questions adapted from previous surveys of Brazilian citizens’ opinions towards science and technology [30, 31] inquiring about their attitudes towards science and technology: “In your opinion, does science and technology bring more damage or more benefits to the world?” (only benefits, more benefits than damage, both benefits and damages, more damage than benefit, only damage) and “If a new technology offers benefits, it should be used even if its’ consequences are not well known” (totally agree, agree in part, disagree in part, totally disagree, do not know).

Participants were then asked to read a short text on male pig castration techniques:

*“The majority of pigs in Brazil are slaughtered at around 5 months of age, as older male pigs begin to sexually mature (e.g. the testicles develop) and there is increased risk that the meat of these animals can express "boar taint." If the pigs are left intact (i.e. with their testicles), approximately 10 to 20% of the meat will express boar taint. Most consumers perceive the taste and odour as very unpleasant. In Brazil, to ensure that meat is not contaminated by boar taint, all male pigs must be castrated prior to slaughter (Decree 9133 of 2017)*.

*The most commonly used technique in Brazil is surgical castration (removal of the testicles). Piglets are castrated between 3 and 10 days of age, usually by the farm staff. The use of medicines to relieve pain is not common in Brazil*.

*An alternative to surgical castration is called immunocastration. The piglets receive two injections with a substance that restricts the development of the testicles. The injection does not contain hormones, but it causes the pig to produce antibodies against its own reproductive hormones. The risk of boar taint in the pigs that have been immunocastrated is eliminated. The method is approved and adopted in several countries, including Brazil*.

*Another alternative is to employ gene-editing technology. This technology makes it possible to alter one gene in the swine embryo. Pigs with this edited gene produce low concentrations of sexual odour in the flesh. Pigs generated using gene edition technology will show this trait. This biotechnology may make castration of piglets unnecessary*.”

Thereafter, participants were asked their opinion regarding the statement, “It is acceptable to produce pork meat using the gene editing technology described above”, with the options ‘totally unacceptable’, ‘unacceptable’, ‘intermediary’, ‘acceptable’, ‘totally acceptable’; thereafter, they were asked to justify their position in an open-ended question. They were then asked to assess the risks (‘no risk’, ‘little risk’, ‘intermediary’, ‘some risk’, ‘high risk’) and benefits (‘no benefits’, ‘little benefit’, ‘intermediary’, ‘some benefit’, ‘high benefit’) associated with the introduction of the gene editing technology described in the text.

Participants were also asked to rate the acceptability of some common biotechnologies used in food production, with response options ranging from 1 (not acceptable) to 5 (very acceptable), and whether they were aware of some pig production practices common in Brazil (“The following statements are true and we want to know if you were aware of this information before filling this questionnaire”, with response options ‘I already knew’ or ‘I did not know’). Finally, the participates were asked to complete a knowledge quiz on biotechnology that had five previously validated questions [16, 24, 32], with response options as ‘true, ‘false and ‘I do not know’.

Statements within the questions regarding acceptability, awareness and knowledge of biotechnologies were randomized. Additionally, for validation purposes we included two check questions where we specifically asked the respondent to mark a given option (e.g., please, for the purpose of validating this questionnaire, mark option 4).

### Data analysis

From the initial 677 participants, 78 were excluded because they were incomplete and an additional 29 were excluded given that participants failed one or both of the check questions resulting in 570 useable questionnaires.

Descriptive statistics for the responses were calculated using Microsoft Excel for Windows and all other statistical analyses were conducted using SAS 9.3. For the question regarding the acceptability to produce pork meat using the gene editing technology, options ‘totally unacceptable and unacceptable’, and ‘acceptable and totally acceptable’ were grouped. Age 56–65 and over 66 years old were also grouped due to the low number of participants in each of these categories.

Spearman’s correlation coefficients were calculated to analyse the degree of association between acceptability of gene editing to prevent boar taint in male pigs and the perception of benefits coming from this technology, the perception of risks, the awareness of common pig production practices, and knowledge of biotechnology assessed in the quiz. The association between the two questions regarding participants attitudes towards science and technology (“In your opinion, does science and technology bring more damage or more benefits to the world?”; “If a new technology offers benefits, it should be used even if its consequences are not well known”) were analysed by Chi-square.

Multinomial logistic regression models were used to analyse associations between acceptability of gene editing to prevent boar taint in male pigs and socio-demographic data. Acceptability of gene editing was considered as a dependent variable. Univariate models were built to separately assess the influence of each predictor variable on the dependent variables. Predictor variables with p < 0.20 were used to build multivariate models. Backward selection was used to eliminate predictor variables until only those with p < 0.10 remained in the models. Results are presented as odds ratio (ODDS) and 95% confidence interval (95% CI). Statistics associations were reported when p ≤ 0.05 and tendency when 0.05 < p ≤ 0.1.

The five-point Likert scale questions about the position of participants regarding acceptability were reclassified into three points (acceptable/ indifferent/ not acceptable).

Data were submitted to thematic analysis, a qualitative method of analysis that allows to capture complexities of meanings in text data, and involves careful reading and rereading of the text for identification of key words, phrases, trends, and themes [33]. Open answers were analysed in three stages: *data reduction* (information is coded), *data display* (organization of the information) and *conclusion drawing and verification* (e.g. triangulation between two or more readers) [34]. To ensure that the coding of themes was appropriate given our study objective, two readers initially analysed 50 random responses and independently developed themes. The two coders (MCY and MJH) then compared their results and discussed any discrepancies and ambiguities until agreement was reached. Finally, the same two readers then coded all answers independently and compared their results and again discussed and reconciled any discrepancies and ambiguities.

## Results

Demographic data are shown in Table 1. Participants’ distribution of sex, age (except for those 66 years old and over), and place of residency approximately corresponded to the Brazilian population according to the Brazilian Institute of Geography and Statistics Census [35] for the three southern states of Brazil. Compared to the general population of the southern region of Brazil, a higher proportion of participants had undergraduate level education and self-identified as not being religious [35].

**Table 1:** Demographics of survey participants (n = 570) and of the associated general population living in southern Brazilians according to latest Brazilian Institute of Geography and Statistics (IBGE) census [35].

Variable	*Participants* *(%)*	IBGE *(%)*
**Sex**		
Female	49	51
Male	51	49
**Age**		
18 to 24 years old	18	16
25 to 34 years old	23	23
35 to 44 years old	25	20
45 to 54 years old	19	18
55 years old and over	15	23
**Education**		
Up to high school	33	64
Undergraduate education (completed or ongoing)	66	36
**Religious**	80	95
**Current residence urban**	95	85
**Involvement with agriculture**		
No involvement	61	
Professional involvement	12	
Grew up in an agricultural environment	27	
**For you, consuming meat is …**		
Important	66	
Intermediate	21	
Not important	10	
**When you eat meat, do you consider the welfare of animals?**		
Always/often	27	
Sometimes	46	
A few times/never	27	

The sources cited were TV (65%), Internet (57%), friends (33%), advertisements of animal food products (24%), university or school (12%), NGO campaigns (10%) and experience with farming (4%). More than half of the participants (57%) had watched a documentary or video on how a pig farm works on the Internet or television and 44% had visited a pig production farm. Fifty percent ate meat 5–7 times a week, 33% ate meat 3–4 times a week, 12% 1–2 times a week and 5% rarely ate meat.

### Acceptability of gene editing to eliminate boar taint

Just over half of participants (56%) considered the gene editing option an acceptable method to reduce boar taint in male pigs, 22% were intermediate and 22% were opposed. Participants that grew up in an agricultural environment had lower odds (ODDS: 0.58, 95% CI 0.36–0.95) of acceptability of gene editing to eliminate boar taint than those that were never involved with agriculture. No other demographic variable, nor awareness of pig production practices or knowledge of biotechnologies influenced the acceptance of male pig gene editing.

### Attitudes towards science and technology

Participants were mostly positive when asked about the benefits of science and technology (Table 2); however, their position was not related to acceptability of gene editing of pigs as a way to prevent boar taint (χ^2^ = 12, 8 df, p < 0.15). Over half of our participants agreed that a new technology that offers benefits should be used despite the downstream consequences not being fully understood (Table 2), and this was associated with acceptability of preventing boar taint using gene editing (χ^2^ = 29.3, df 8, p < 0.001).

**Table 2:** Participants position on damages and benefits of science and technology and their position on whether the technology should be used despite no available knowledge regarding its long-term consequences (n = 570).

	Participants	Southern region [31]*
**In your opinion, does science and technology bring more damage or more benefits to the world?**		
Only benefits	16%	51%
More benefits	41%	30%
Equal benefits & damage	41%	7%
More damage	2%	3%
Don’t know/didn’t answer	0%	9%
Only damage	0%	0%
**If a new technology offers benefits, it should be used even if its consequences are not well known?**		
Totally agree	7%	13%
Partly agree	48%	21%
Partly disagree	24%	19%
Totally disagree	19%	40%
Don’t know	2%	7%

*The Centre for Management and Strategic Studies (CGEE) is a social organization supervised by the Ministry of Science, Technology, Innovation and Communications (MCTIC). The 2015 survey on public perception of science and technology in Brazil aimed to discover how much Brazilian population knows about issues related to the area.

### Perception of risks and benefits of gene editing of pigs to prevent boar taint

Most participants (65%) perceived some or numerous benefits from the use of gene editing of pigs to prevent boar taint, 22% were intermediate and 13% perceived little or no benefits. The acceptability of gene editing to prevent boar taint in male pigs was positively related to the perception of benefits coming from this technology (r = 0.56, ρ <0.0001).

Thirty nine percent of participants perceived much or some risk from the implementation of this technology, 35% perceived little or no risk, and 26% positioned themselves as intermediate. The acceptability of gene editing to prevent boar taint in male pigs was negatively related to the perception of risks (r = -0.35, ρ <0.0001).

### Acceptability of biotechnologies in food production

Acceptability of different application of biotechnology to produce food was in general low (Table 3).

**Table 3:** Acceptability of different plant and animal-based biotechnologies used in food production of southern Brazilian residents’ participants (n = 570).

	Acceptable	Intermediate	Not Acceptable
Vegetables genetically modified to contain higher concentrations of nutrients for food	43%	20%	37%
Microorganisms genetically modified to improve their efficiency for production of fermented food for human consumption	31%	20%	44%
Pigs genetically modified to produce more meat for human consumption	28%	18%	54%
Meat produced by pigs fed with diets containing transgenic components	28%	26%	52%
Meat produced *in vitro* from pig stem cells	25%	20%	55%

### Awareness of pig production issues

When asked specifically about pig production practices in Brazil, participants were mostly unaware (Table 4). Acceptability of gene editing to prevent boar taint in male pigs was not related to awareness of common pig production practices (r = 0.032, ρ < 0.4).

**Table 4:** Participants’ awareness on six pig production issues (n = 570 southern Brazilian residents).

Question	Awareness (% participants)
Most pigs and poultry feeds used in Brazil are produced with transgenic soy and corn	71
Genetic modifications can be induced in animals through biotechnologies to improve various characteristics, such as heat resistance, protein production or disease resistance	54
Surgical castration of pigs without pain control is the most common technique in Brazil	30
Meat from non-castrated pigs slaughtered after puberty may present boar taint	29
All male pigs slaughtered in Brazil are castrated	24
Vaccines used to stimulate the body to produce antibodies do not leave residues in animal products	20

### Knowledge of genetics and biotechnology

Few participants answered the questions on the biotechnology knowledge quiz correctly (Table 5). Acceptability of gene editing to prevent boar taint in male pigs was not related to knowledge of biotechnology assessed in the quiz (r = 0.062, ρ<0.14).

**Table 5:** Percentage of correct, incorrect and do not know answers of southern Brazil residents (n = 570) on the biotechnology knowledge quiz*.

Question	% correct	% incorrect	% don’t know
By eating a genetically modified food, a person’s genes could also become modified	57	10	33
Pigs modified with genes from a fish would probably taste fishy	43	5	52
Ordinary tomatoes do not contain genes, while genetically modified tomatoes do	39	13	48
Genetically modified animals are always bigger than ordinary ones	36	28	36
It is possible to transfer animal genes into plants	14	30	56

*Questions were adapted from [16, 24, 32].

### Participants’ reasons to justify the acceptability of gene editing of pigs to prevent boar taint

Table 6 shows the proportion of participants with different positions regarding the acceptability of gene editing of pigs to prevent boar taint. The main themes used by participants to justify their position were in descending order: 1) positive effects on animal welfare (42%), 2) doubts or perception of potential risks of gene editing (31%), 3) perceived effects on product quality (11%), 4) insufficient information on the issue to form an opinion (9%), 5) dislikes or is opposed to genetic modification (7%), and 6) perceives gene editing as unnatural (4%). Of the total survey participants that mentioned risks, 38% mentioned potential risks to humans, 27% risks to animals, and 35% unknown or unspecified risks. Regarding meat quality, participants perceived both positive and negative effects.

**Table 6:** Reasons presented by participants to justify their position regarding the use of gene editing technology to reduce boar taint in pigs. Data are shown as % participants in each group*.

	Position regarding gene editing to reduce boar taint			
Themes associated with justifying position	Total**	Acceptable	Intermediate	Not acceptable
Positive effects on animal welfare	45%	63%	19%	16%
Potential risks of gene editing	34%	26%	61%	25%
(to humans)	(13%)	(11%)	(23%)	(6%)
(to the animals)	(9%)	(6%)	(17%)	(11%)
(not specified)	(10%)	(10%)	(21%)	(8%)
Perceived effects of product quality	11%	10%	16%	11%
Insufficient information on the issue	10%	5%	27%	8%
Dislike or opposition to genetic modification	8%	1%	2%	38%
Gene editing is unnatural	5%	3%	2%	14%

*Percentages shown in the table were calculated considering the participants that answered the open question: 75%, 75% and 59% of the participants that considered gene editing acceptable, intermediate and not acceptable, respectively. Note that participants’ answers could cover more than one theme, therefore do not add to 100%.

**The % of all respondents who raised each theme

Participants’ acceptance was often (63%) related to the belief that castration causes pain and suffering, which was seen as not acceptable: *It is interesting*, *given that it reduces animal suffering* (M084 Position = Acceptable); *To end animal suffering* (M038 Position = Acceptable); *It is acceptable because this way pig castration won’t be necessary*, *preventing them from experiencing the pain* (R081, Position = Acceptable); *I believe that a technology that does not cause suffering to animals and does not incorporate foreign (unnatural) substances into the metabolism of animals must be used* (M261 Position = Acceptable). It was suggested that consumers would value the positive effects on animal welfare when faced with products from gene edited animals: *Anything that is for animal welfare is very valid and important*, *and surely the consumer will take this into account when they realise that animals are produced in this way* (with gene editing) (M127, Position = Acceptable).

A quarter of the participants that found the technology acceptable expressed concerns with the unknown effects of the technology: *The genetic mutation to which pigs would be subjected is as unnatural and inhuman as castration*. *However*, *I believe that the gene-editing* {alternative} *is less harmful or barbaric* (R135, Position = Acceptable); *Any genetic change is troubling*. *What kind of side effect may it have*? *But to avoid animal suffering*, *then I prefer it* (R154, Position = Acceptable).

Some weighed the acceptability of the technology in terms of the ‘lesser of two evils’, often referencing animal suffering and meat contamination, especially by hormones:

If all the tests are done and it does not bring any risks, I agree, since we already eat a lot of contaminated meat. For everything that represents animal suffering, if there is a way for it not to happen, then it is acceptable

(R052, Position = Intermediate)

Animals would not be subjected to unnecessary torture and it would reduce concerns about the vaccines used for immunocastration

(M257, Position = Acceptable)

The less suffering for the animal the better and it does not need to undergo the transformation of the meat with antibodies produced against its own hormones

(M208, Position = Acceptable)

*I believe this is the most practical* (alternative), *without the suffering caused by surgical procedures and without the risk of any effects or modifications caused by hormonal applications*

(M225, Position = Acceptable)

Other participants concluded that the potential offered by the technology to improve animal welfare does not justify subjecting the animals to genetic modification. Some weighed the potential risks of the technology (*I do not trust genetic manipulation as a mechanism to improve animal welfare*. *I think this alternative can’t be compared to the others because the long-term consequences are unknown*; R110, Position = not acceptable), and others expressed moral considerations (*Although it doesn’t cause pain to the animal*, *it’s sad to know that the pig will be genetically modified in order to be consumed*, R050, Position = not acceptable).

Scientific evidence in support of no harmful effects was expected by many (34%) as a condition for acceptance of the technology; this was often associated with concerns with potential risks for humans (e.g., *Because we are not sure of the development of a human being that consumes such a modified animal*, *whether it can generate new diseases*, *or not*. *It could be fit for testing*, *but not for immediate use*, M078, Position = Not acceptable; *How sure are we that this biotechnology will not affect humans*? *If there were studies proving that this will have no side effects on those who ingest this meat*, *then I would support it*, M270, Position = Intermediate; *I have doubts regarding consumption and the transmission to humans at the genetic level*, 006, Position = Acceptable), the animals (e.g., *My doubt is whether this gene editing may cause genetic erosion within the pig population*, M270, Position = Intermediate), or both (e.g., *Gene editing is totally acceptable as long as there’s no side effects or risks of hormonal or genetic changes*, *as much in the pig as in the consumer*, R170, Position = Acceptable). However, a third of those expressing concerns with potential risks of harm stated this in general terms, without specifying the kind of harm (e.g., *I think that with the advancement of biotechnology many problems can be solved*, *but we must consider the risks that this DNA modification may end up generating new problems*, R266, Position = Acceptable; *Long-term studies are needed to show that this change*, *or the gene in general*, *are not harmful*, R209, Position = Intermediate; *I am highly suspicious regarding genetic modification*. *It needs to be thoroughly studied so that we don’t suffer the consequences later*, M270, Position = Intermediate).

Among the implications of the introduction of the technology proposed, some participants discussed indirect effects on the producers: *The text implies that this way of castration is not painful for the animal* … *But one reservation is related to the cost of this technology for the producer*, *already subjected to hardship by the economic ups and downs*; R147, Position = Acceptable). Others commented on implications for the industry, with some participants implying that the introduction of the technology could lead to an increase in the power of larger corporations to the detriment of smaller producers: *Another issue is the question of costs*, *I know that small producers usually make little money from livestock production*, *wouldn’t the cost increase*? *The big meat packing companies could afford the increased costs*, *but how about the small guys*? M119, Position = Acceptable).

Another concern expressed was the distrust that parties interested in developing the technology would genuinely be interested in improving animal welfare: *Somewhat acceptable*, *because I need more clarification regarding the issue*. *In general*, *the companies that own this kind of knowledge care more about profits than about the natural environment*, *the animals and consumers’ health* (R258, Position = not acceptable); *I think it is fair if we consider that it may cause the least possible suffering to the animal; however*, *I do not believe that the use of this technology is aimed at the welfare of the animal*, *rather at potential greater financial returns* (R176, Position = Acceptable).

Many participants that were unsure in their position (27%), amongst others, claimed that they needed more information to give an opinion (e.g., *It’s difficult to understand all this process from a single paragraph explaining the issue*. *Anything that involves genetic manipulation deserves to be carefully studied*, *for ethical reasons*, M088, Position = Intermediate; *I don’t know the technology*, *which is for me a limiting factor for giving an opinion*, M051, Position = Intermediate; *Does the gene issue only have good points*? *I don’t have enough information to say that it’s totally acceptable*, M119, Position = Acceptable)

Some participants suggested that the sources of information available to them may lack transparency: *I didn’t know about surgical castration*, *that is something the ads don’t show*, R030 (Position = Acceptable); *…because it makes castration unnecessary; however*, *there may be some negative aspects yet unknown (and perhaps already expected)* (R088, Position = Acceptable); *I don’t know the gene editing method and I don’t have conditions to assess it just from the information above*, *there may be consequences not informed there*, M073, Position = Intermediate.

Loss of naturalness was discussed primarily by participants that stated that gene editing was not acceptable (14%) (*I don’t like using science to manipulate nature*. *I believe that the excessive exploitation of animals is a mistake*. *We must respect nature and preserve it*. *This action does not preserve it*, M133, Position = not acceptable; *It goes against the ‘natural law’ of the animal*, *of how it should happen* …, R065, Position = not acceptable; *I have insufficient information to give an opinion*. *But I find this too artificial* (M245, Position = not acceptable). However, naturalness was also mentioned by some participants that indicated acceptance of gene editing, for example: *The pigs would not feel pain*, *but it would not be something natural* (M056, Position = acceptable).

Opposition to gene editing to eliminate boar taint was in large part (38%) associated with an objection, usually of moral nature, to any kind of genetic modification: *I find it outrageous to change the genetic make-up of a living being simply so that it meets a ‘need’ in the consumer market*, R059, Position = not acceptable; *In my opinion anything that needs to be modified*, *shouldn’t be eaten*, R190, Position = not acceptable; *Even being ‘scientific’*, *I do not agree with these changes*, R025, Position = not acceptable; *I don’t like to change anything genetically*, M093, Position = not acceptable). Some participants articulated pragmatic arguments regarding the potential of unknown consequences to explain their opposition to genetic modification: *I don’t agree with genetic modification of animals for consumption*. *I don’t believe that all the consequences are well known*; R044, Position = not acceptable; *The more we use genetic experiments*, *the more prone to mutations we are*, *because they* (referring to the “experiments”) *are not 100% safe*; M280, Position = not acceptable).

## Discussion

Just over half of the participants found the scenario of gene edited male pigs to prevent boar taint acceptable. In general, acceptance was justified by perceived improvements in animal welfare; rejection, in contrast, was related to opposition to genetic modification and perceived loss of naturalness. Equally important, however, was that acceptance was often conditional either directly or indirectly, with participants stating that they desired: 1) assurance that unforeseen harm to humans, animals and the environment would be prevented, and 2) greater clarification of the process and its consequences.

### Attitudes towards animal suffering

Reducing animal suffering was the main issue discussed by participants that supported the use of gene editing in pigs. Generally, the participants of this survey were critical of pain involved in surgical castration, despite low awareness regarding Brazilian pig production practices. Pain caused by human intervention is one of the most important farm animal welfare concerns among lay citizens [36, 37]. More specifically, European citizens show negative attitudes towards piglets’ castration without anaesthesia [38, 39], which has led Europe to discuss steps to ban the practice [6]. As previously shown in Europe [40, 41] most participants were not aware of the widespread use of surgical castration in Brazil, but many considered it cruel or negative for the welfare of the animals. Others have shown that lay citizens’ low awareness of livestock production systems and practices does not explain negative attitudes towards contentious practices [42–44]. Our findings suggest that perceived benefits to the welfare of animals are likely to reduce the relative importance given to perceived risks, resulting in increased support of new technologies. This supports similar conclusions of [45], who surveyed North Americans’ attitudes regarding the use of GM to produce polled cattle. Whether this also applies to other gene editing applications that may reduce animal suffering, such as heat stress [28, 46], remains to be seen. Also, considering that support to gene editing was largely based on the perception that it may improve animal welfare, to maintain public trust the implications of gene editing on animal welfare should be made transparent to the public. For example, those associated with biotechnologies involved in the process of embryo transfer [47].

### Perceptions of risks of genetic modification of food animals

It is perhaps equally relevant that supporting, opposing and undecided participants raised numerous concerns regarding potential risks arising from gene editing. Participants opposing the technology presented these risks as a reason for failure to accept, but many were undecided. Supporting participants frequently demanded clarification or assurance against their concerns as a condition to full acceptance. Gene editing is presented as more efficient and safer compared with previous genetic engineering methods [12, 48]. However, the safety of this technology is being questioned by some [49]. Also, some authors recognise the potential of the technology but warn that the potential risks to animals, humans, the environment and society must not be overlooked [28, 50–52]. Concerns with unknown or only partially known outcomes were conveyed by our participants and encompassed issues like future harm to animal and human health–such as creation of new diseases and undesired mutations–harm to the species’ integrity and animal welfare, loss of naturalness, and environmental hazards.

It has been argued that the largest factor influencing acceptability of gene editing in agriculture may be the perceived usefulness of the application to humans [53] and, more specifically, the perceived benefits to consumers as opposed to the industry [54, 55]. Participants, especially those unsure about the application, presented concerns about biological and societal risks. Thus, stakeholders seeking social support for these applications need to prioritize communication on risks perceived by the public [56–58]. The topic of gene edition has a growing presence in the media, and it is often reported as a novel technology able to solve important problems; however, to date science cannot provide all answers about the genetic background of traits involved or how it may work once in large-scale use [28], issues that have not been clearly conveyed to the public. Importantly, the vehicles for communication need to be carefully chosen, as trust in information sources about new technologies hinges, among other things, on credibility and shared values with providers of information [59, 60]. Also, it is important to consider that lay people tend to assess the risks of technologies and the acceptability of such risks differently from specialists [60].

Concerns regarding societal risks of the use of the gene editing application in food animal production were also expressed by participants. For example, some of our survey participants questioned the motivation of proponents of food animal gene editing, arguing that large animal industries seek economic gains rather than improving animal welfare, or arguing whether small producers would have access to it. Similarly, negative attitudes towards GM crops among Brazilian lay citizens was related to perception of few benefits for consumers and family farmers, as opposed to large producers [61]. A common concern regarding the application of biotechnologies in agriculture are the social consequences for small producers or breeders that may become unable to compete with larger corporations [27, 62, 63]. Concerns with the consequences of the introduction of costly biotechnologies for family farms in Brazil may be relevant, as smallholder family farmers are responsible for over 70% of the food consumed domestically, which brings food security and social wellbeing considerations [64, 65]. Concern shown here, and also in [45], for equity of access and the commercialization of genome editing technologies are largely absent from the academic debate [66]. Finally, some concerns raised by participants warn about the need to discuss with society the implications of the use of technologies like gene editing for the future of food animal production, in the wider context of sustainability of livestock production [67, 68].

### Role of knowledge and information on attitudes

Our participants had little knowledge of basic biotechnology concepts, a fact shown by others [16, 69–71]. In contrast to others who reported that individuals more knowledgeable of basic genetics and biotechnologies are more critical of biotechnologies [16, 71], level of knowledge was not associated with acceptability of gene editing of pigs. Mielby et al. [15] warned that conclusions concerning the effect of knowledge on acceptance of GM technologies cannot be generalized to all applications. Whereas risk perception is more influenced by scientific literacy [15], perception of benefits for animal welfare, which are related to moral values rather than knowledge or awareness [43, 72], may enhance acceptance of risks of using gene edition in animals. Further support for this conclusion was the lower acceptance by our participants of other uses of biotechnologies used in livestock production, such as feeding pigs with diets containing transgenic components and genetically modifying pigs to produce more meat for human consumption.

Participants complained about the lack of information available to them regarding the risks of GM technologies, some believing that the risks of the use of genetic engineering in food production are hidden from society. In a survey on perceptions towards transgenic crops [61] Brazilian lay citizens expressed a similar discontentment with information sources and complained that they had been misinformed, which caused confusion and distrust. Media reporting of genetic modification is characterized by a large volume of information of varying levels of accuracy and types of content; contradictory messages from different sources and actors in the risk debate, ‘dramatization’ of risk information though ‘scenarios’, and the symbolic connotations of terms or concepts used in messages may contribute to the public concern with risks [55]. For example, two biotechnologies used in animal production that participants perceived as less acceptable than gene editing to eliminate boar taint–the use of diets containing transgenic components and artificial meat–have both received considerable media coverage in Brazil (e.g., [73, 74]. In contrast, given the level of awareness of our participants it is likely that many were introduced to the subject of gene editing of food animals for the first time when taking our survey. People use mental short-cuts to facilitate and speed up the decision-making process [75], accessing the pool of feelings, either positive or negative, that they associate with the issue in question [76]. Thus, participants that related the information offered in the text to benefits to the animals may have been influenced to choose the option “acceptable”, and those who related it to moral concerns regarding gene manipulation and future harm to human and animal health may have chosen the option “not acceptable”. Another study found that doubts and knowledge gaps concerning the potential risks for human health and the environment arising from the use of transgenic crops for human food were often translated by survey participants into distrust towards the technology [77].

Gene edition to produce pigs that do not express boar taint is an application that is still in its preliminary stages of development [12, 78, 79] and thus our study is timely. It has been argued that it is desirable to understand public’s views and acceptability before a new technology is introduced [22]. Questions regarding GM and genome editing should also be discussed with the public in the context of modern farm animal breeding, and not as a separate phenomenon [28]. In this context, we explained to survey participants that castration of male pigs in Brazil is mandatory and presented the gene edition option as an alternative to surgical castration and immunocastration, two widely used practices on farms in Brazil. The fact that the participants in our study were provided information that essentially forced them to trade off gene editing with immunocastration or surgical castration, which they were told was painful without anaesthesia, may have influenced the acceptability rates found in this survey. The qualitative responses enabled us to determine that indeed most participants did compare the proposed GM method to the alternatives presently used by the industry, despite the fact that they had little prior knowledge of any of the practices. This finding is encouraging as it does provide evidence that the lay public is capable of engaging in meaningful discussion on specific agricultural practices.

### Limitations of the study

Our sample of participants was more highly educated, matched age, sex and religiousness of the region where the survey was carried out. Nation-wide surveys undertaken in 2010 and 2015 [31] have shown that Brazilian citizens have low knowledge but positive attitudes towards science and technology, a finding that was confirmed in our sample; importantly, these surveys showed that education, access to information, income, region of residence within the country, and interest in science and technology do not influence these attitudes [30]. This supports our findings that demographic characteristic did not influence the acceptability of gene edition. Growing up in agricultural environment was the only demographic characteristic that reduced acceptability. However, it is important to note that our participants had a great proportion of people with university education, and thus not representative of the Brazilian population.

## Conclusions

The participants surveyed in this study placed great value on the potential animal welfare benefits arising from gene editing to remove boar taint in pigs, citing this as their primary reason for supporting this practice. However, this support was not unconditional but rather was accompanied by responses that users of these types of technologies must be clear, honest and transparent in their communications regarding the unknown downstream risks associated with the technology. Perceived risks and uncertainty may be more determinant of public attitudes towards gene editing of farm animals, particularly in the case of applications that do not involve improvement in animal welfare.

## Supporting information

S1 Table Survey questions. (XLSX)

S2 Table Type 3 analysis of effects based on multinomial logistic regression. (XLSX)

S1 Data Questionnaire responses. (XLSX)
